# Discordance Between Patient-Reported Outcomes and Heel-Rise Limb Symmetry After Surgical Repair of Acute Achilles Tendon Rupture

**DOI:** 10.3390/jcm15124669

**Published:** 2026-06-16

**Authors:** Firat Dogruoz, Aydogan Askin, Ali Ergun, Emre Mucahit Kartal, Serkan Gurcan, Ozkan Kose

**Affiliations:** 1 Department of Orthopedics and Traumatology, University of Health Sciences, Antalya Education and Research Hospital, 07100 Antalya, Turkey; firatdogruoz@hotmail.com (F.D.); aydoganaskin1@gmail.com (A.A.); a_ergun83@hotmail.com (A.E.); 2 Department of Orthopedics and Traumatology, Antalya City Hospital, 07070 Antalya, Turkey; 3 Private Practice, Gurcan Orthopedics Clinic, Cevizlik, Bakırköy, 34365 Istanbul, Turkey

**Keywords:** Achilles tendon rupture, Achilles tendon repair, patient-reported outcome measures, limb symmetry index, heel-rise test, functional recovery, calf muscle endurance

## Abstract

**Background/Objectives:** The extent to which patient-reported outcome measures (PROMs) reflect objective functional recovery after surgical repair of acute Achilles tendon rupture remains unclear. This study aimed to evaluate the relationship between PROMs and objective functional performance, particularly the heel-rise test-based limb symmetry index (LSI). **Methods:** This retrospective cohort study included male patients who underwent primary open repair for acute Achilles tendon rupture between 2015 and 2023 and had a minimum follow-up of 12 months. Subjective outcomes were assessed using the Achilles Tendon Total Rupture Score (ATRS) and the American Orthopaedic Foot and Ankle Society (AOFAS) Ankle–Hindfoot Score. Objective functional performance was evaluated using a single-leg heel-rise test, and LSI was calculated as the percentage performance of the injured limb relative to the uninjured limb. Patients were stratified into two groups according to LSI: ≥90% and <90%. Correlations between PROMs and LSI were analyzed using Spearman’s rank correlation coefficient. **Results:** A total of 76 male patients were included. The median age was 42 years (IQR, 35–46; range, 27–74), and the median follow-up duration was 41 months (IQR, 26–53; range, 12–80). The median ATRS was 97 points (IQR, 94–99), and the median AOFAS score was 98 points (IQR, 90–100). The median LSI was 87.5% (IQR, 81.4–93.3), and only 31 patients (40.8%) achieved an LSI ≥ 90%. Injured-side heel-rise performance was significantly higher in the LSI ≥ 90% group than in the LSI < 90% group (24.4 ± 5.4 vs. 21.6 ± 5.9 repetitions, *p* = 0.039). However, ATRS and AOFAS scores did not differ significantly between the groups. No significant correlation was found between LSI and either ATRS (Spearman’s rho = 0.190, *p* = 0.100) or AOFAS score (Spearman’s rho = 0.218, *p* = 0.058). **Conclusions:** Although PROM scores were high following surgical repair of acute Achilles tendon rupture, objective functional assessment revealed persistent limb-symmetry deficits in a substantial proportion of patients. ATRS and AOFAS scores showed no significant correlation with heel-rise LSI, suggesting that PROMs may not fully capture objective functional recovery. Therefore, PROMs should be interpreted alongside performance-based measures, particularly in patients with high functional demands.

## 1. Introduction

The Achilles tendon plays a critical role in lower-limb biomechanics by enabling efficient force transmission during activities such as walking, running, and jumping. Acute Achilles tendon rupture (ATR) is a common injury, particularly among physically active adults, and its incidence has increased in recent decades [1]. Given the Achilles tendon’s functional importance, accurate assessment of recovery after treatment remains a key clinical priority.

Outcome evaluation after ATR repair traditionally relies on both patient-reported outcome measures (PROMs) and objective functional assessments [2,3]. PROMs, particularly the Achilles tendon Total Rupture Score (ATRS), are widely used because they capture patient-reported symptoms, function, and satisfaction [4]. The American Orthopaedic Foot and Ankle Society (AOFAS) Ankle–Hindfoot Score is also frequently used in clinical practice, although it has recognized limitations, including potential ceiling effects and clinician-reported components [5,6]. Because ATRS is an Achilles tendon-specific PROM, whereas AOFAS is a more general ankle–hindfoot score with both patient- and clinician-reported components, evaluating both scores may provide a broader understanding of how commonly used clinical outcome measures reflect recovery after ATR repair.

Despite their widespread use, PROMs may not fully reflect objective functional recovery [2]. Objective measures such as heel-rise performance, muscle endurance, calf circumference, and ankle range of motion provide quantifiable data on physical recovery and may reveal persistent deficits that are not captured by subjective scoring systems [2,7]. In particular, the heel-rise test and the derived limb symmetry index (LSI) have been shown to be practical and reliable tools for evaluating calf muscle function after ATR [7,8]. This distinction is clinically relevant because patients may report satisfactory symptoms and function while still demonstrating measurable deficits in calf endurance, limb symmetry, or ankle mobility. Such discrepancies may influence rehabilitation planning, return-to-activity decisions, and the interpretation of treatment success.

However, the relationship between PROMs and objective functional outcomes remains incompletely understood. Existing studies investigating this association are limited in number and have reported inconsistent findings, with some demonstrating weak correlations and others showing no meaningful relationship between subjective and objective recovery parameters [8,9]. Furthermore, the extent to which commonly used PROMs reflect functional symmetry and performance-based recovery remains unclear [9,10]. Therefore, further evaluation of the agreement between patient-perceived recovery and objective functional performance is needed to determine whether PROMs alone are sufficient for postoperative assessment after ATR repair.

Therefore, the primary aim of this study was to evaluate the relationship between PROMs (ATRS and AOFAS) and objective functional recovery, as measured by the heel-rise test-based limb symmetry index (LSI), in patients who underwent surgical repair for acute Achilles tendon rupture. The secondary aim was to assess additional objective parameters, including calf muscle atrophy and deficits in ankle range of motion. We hypothesized that PROM scores would show a weak or absent correlation with objective functional performance, suggesting a potential discrepancy between patient-perceived and actual recovery.

## 2. Materials and Methods

### 2.1. Patients and Study Design

This retrospective cohort study was conducted at a tertiary referral university hospital and included patients who underwent surgical repair for acute Achilles tendon rupture (ATR) between January 2015 and December 2023. The diagnosis of ATR was established based on clinical examination findings, including a positive Thompson test and a palpable tendon gap, and was confirmed with ultrasonography or magnetic resonance imaging when necessary. The indication for surgical treatment was based on a standardized institutional protocol that considered patient-related factors such as age, activity level, and functional demands. In general, surgical treatment was preferred for physically active individuals and patients with higher functional expectations. All procedures were performed by experienced orthopedic surgeons using the same open repair technique, and all patients followed a standardized postoperative rehabilitation protocol.

Patients were first identified retrospectively from hospital records. Eligible patients were then invited to attend a final clinical and functional evaluation at least 12 months after surgery. Patients who could not be contacted, declined to participate, had a follow-up duration of less than 12 months, were unable to complete the physical examination and functional testing, or had incomplete clinical or functional assessment data were excluded. Additional exclusion criteria included chronic ruptures (>4 weeks), additional procedures such as tendon transfers, prior surgery or injury affecting the contralateral lower extremity, bilateral sequential Achilles tendon rupture, and re-rupture requiring revision surgery. After the eligible cohort was identified and invited to the final assessment, female patients were excluded from the final analysis because they represented only a very small proportion of the cohort (*n* = 3). Their inclusion would not have permitted a meaningful sex-based subgroup analysis and could have introduced additional variability into objective functional measures, as sex-related differences may influence calf circumference, muscle strength, heel-rise performance, and limb symmetry. Therefore, the final analysis was restricted to male patients to obtain a more homogeneous study population for evaluating the relationship between PROMs and heel-rise LSI.

Ethical approval was obtained from the institutional review board, and all participating patients provided written informed consent prior to the final evaluation (Approval Date: 23 October 2025, No.:18/7). This study was conducted in accordance with the Declaration of Helsinki and reported in line with the STROBE guidelines.

### 2.2. Sample Size Calculation

The sample size was calculated for the primary analysis, defined as the correlation between the Achilles tendon Total Rupture Score (ATRS) and the heel-rise test limb symmetry index (LSI). Based on previously published data by Cramer et al., who reported a moderate correlation (r = 0.35) between patient-reported outcomes and objective functional performance following Achilles tendon rupture, a two-tailed correlation test was used, with a significance level (α) of 0.05 and a statistical power (1 − β) of 0.80 [8]. Accordingly, a minimum of 58 patients were required to detect a correlation of similar magnitude. Although the sample size estimation was based on a Pearson correlation framework, the final correlation analyses were performed using Spearman’s rank correlation coefficient because the observed data, particularly the PROM scores, were not normally distributed and demonstrated a marked ceiling effect. Therefore, a sample size calculation was used to estimate the required cohort size to detect an anticipated correlation effect, whereas the final correlation method was selected based on the distributional characteristics of the collected data.

### 2.3. Surgical Technique and Postoperative Rehabilitation

The same surgical team performed all surgical procedures. Although more than one surgeon was involved, all operations were conducted using a standardized open repair technique in accordance with the institutional protocol. Patients were positioned prone under spinal or epidural anesthesia, and a pneumatic tourniquet was used in all cases. A posterior midline incision was made to expose the ruptured tendon. Primary end-to-end repair was performed using a Kessler-type core suture technique with No. 5 non-absorbable sutures. No routine augmentation procedures, such as tendon transfer or graft reinforcement, were performed. The paratenon was carefully closed in all cases. The rupture level was typically in the mid-substance of the tendon, and patients with insertional or musculotendinous-junction ruptures requiring modified repair techniques were excluded.

Postoperative management followed a standardized institutional rehabilitation protocol that remained consistent throughout the study period. Immediately after surgery, the ankle was immobilized in approximately 5–10° of plantar flexion using a short-leg splint. Patients were mobilized with crutches, without weight-bearing, for the first 4 weeks. At 4 weeks postoperatively, the splint was removed, and supervised physiotherapy was initiated, including gradual passive and active range-of-motion exercises, with avoidance of forced dorsiflexion. Partial weight-bearing was introduced progressively after 6 weeks, followed by strengthening exercises. This relatively conservative rehabilitation protocol was adopted to minimize the risk of tendon elongation and re-rupture, particularly during the early healing phase [11,12,13]. The protocol was applied uniformly to all patients to ensure consistency in postoperative management. Return to sports activities was generally permitted after the sixth postoperative month, based on clinical assessment, including pain-free range of motion, adequate strength recovery, and functional performance during rehabilitation [13].

### 2.4. Functional Evaluations and Outcome Measures

All final assessments were performed by a single independent assessor with experience in musculoskeletal evaluation, who was blinded to the patient-reported outcome scores to minimize measurement bias. All measurements were conducted according to a standardized protocol. Subjective outcomes were evaluated using the Achilles tendon Total Rupture Score (ATRS) and the American Orthopaedic Foot and Ankle Society (AOFAS) Ankle–Hindfoot Score [4,5]. The same assessor administered the AOFAS score during the clinical evaluation.

Objective functional performance was assessed using a standardized single-leg heel-rise endurance test [7,14]. Patients performed the test barefoot while standing on one leg, with light fingertip support against a wall for balance. A metronome was used to standardize the pace at 30 heel rises per minute. Each repetition was considered valid only if the patient achieved full plantar flexion, comparable to the contralateral side. The test was terminated when the patient was unable to maintain the required pace, failed to reach adequate heel height, or voluntarily stopped due to fatigue. To reduce potential learning or order effects, the starting limb (injured or uninjured) was alternated between patients.

The number of valid repetitions for each limb was recorded. The limb symmetry index (LSI) was calculated as the ratio of the injured side to the uninjured side and expressed as a percentage [10,15]. Given that the test was based on repetition count rather than heel-rise height, this parameter was defined as an endurance-based limb symmetry index.

Calf circumference was measured bilaterally at the point of maximal girth in a relaxed standing position, and the interlimb difference was recorded as an indicator of muscle atrophy. Ankle range of motion was measured using a standard goniometer, with the patient supine and the knee extended. Dorsiflexion and plantar flexion angles were recorded for both limbs, and side-to-side differences were calculated. Postoperative complications, including wound problems, infections, re-ruptures, and neurological deficits, were systematically recorded during follow-up.

### 2.5. Statistical Analysis

Descriptive statistics were presented as mean ± standard deviation for normally distributed continuous variables and as median with interquartile range for non-normally distributed continuous variables. Minimum and maximum values were also reported where appropriate. Categorical variables were summarized as frequencies and percentages. The normality of continuous variables was assessed using the Shapiro–Wilk test. Patients were stratified into two groups based on the heel-rise limb symmetry index (LSI). A heel-rise LSI threshold of ≥90% was used to define sufficient functional recovery, as this cutoff has commonly been used in previous lower-extremity functional recovery and Achilles tendon repair studies as a practical benchmark for acceptable limb symmetry [10,15]. This threshold was not intended to represent a definitive biological marker of complete tendon healing, but rather a clinically interpretable reference value for comparing patients with relatively sufficient and insufficient functional symmetry. Patients with an LSI ≥ 90% were classified as having sufficient functional recovery, whereas those with an LSI < 90% were classified as having insufficient functional recovery. Comparisons between the two groups were performed using the independent-samples *t*-test for normally distributed continuous variables and the Mann–Whitney U test for non-normally distributed continuous variables. Fisher’s exact test was used for categorical variables when appropriate. Correlations between patient-reported outcome measures and objective functional parameters were evaluated using Spearman’s rank correlation coefficient because several continuous variables, particularly the PROM scores, were not normally distributed and demonstrated ceiling effects. In addition, a multivariable linear regression analysis was performed to determine whether patient-reported outcome measures were independently associated with heel-rise LSI. LSI was entered as the dependent variable. ATRS and AOFAS scores were entered as independent variables together with clinically relevant covariates, including age, BMI, and follow-up duration. Regression coefficients, along with 95% confidence intervals and *p*-values, were reported. Statistical significance was set at *p* < 0.05. Missing or incomplete data were handled using a complete-case approach. Patients with incomplete demographic, clinical, PROM, or functional assessment data were excluded before analysis. Therefore, no data imputation or additional sensitivity analysis for missing data was performed.

## 3. Results

**Figure 1:**
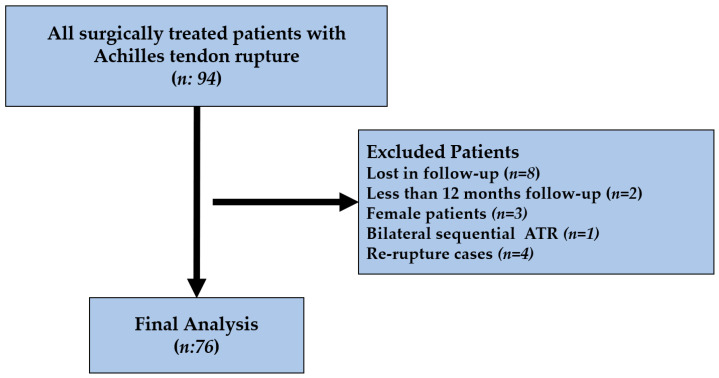
Flowchart demonstrating patient selection and exclusion criteria.

A total of 94 patients who underwent surgical treatment for acute Achilles tendon rupture were initially identified from hospital records. Of these, 18 patients were excluded because of loss to follow-up (*n* = 8), follow-up duration of less than 12 months (*n* = 2), female sex (*n* = 3), bilateral sequential Achilles tendon rupture (*n* = 1), or re-rupture requiring revision surgery (*n* = 4). Ultimately, 76 male patients were included in the final analysis. The patient selection process is shown in Figure 1.

The median age was 42 years (IQR, 35–46; range, 27–74 years). The right side was involved in 39 patients (51.3%), and the left side in 37 patients (48.7%). The median follow-up duration was 41 months (IQR, 26–53; range, 12–80 months). Postoperative complications included superficial wound infection in six patients (7.9%), all of which were managed successfully with oral antibiotics and local wound care. Sural nerve-related sensory disturbances were observed in two patients (2.6%) and were considered persistent based on clinical evaluation at the final follow-up. A reduction in ankle range of motion compared with the contralateral side was observed in 12 patients (15.7%). Quantitatively, the median dorsiflexion loss was 0° (IQR, 0–0; range, 0–10°), and the median plantar flexion loss was 0° (IQR, 0–0; range, 0–20°). The median ATRS was 97 points (IQR, 94–99; range, 71–100), and the median AOFAS score was 98 points (IQR, 90–100; range, 70–100). Calf muscle atrophy was observed in 64 of 76 patients (84.2%), with a median side-to-side difference of 1.0 cm (IQR, 1.0–2.0; range, 0–10 cm) in calf circumference between the injured and uninjured limbs. Based on the heel-rise test, the intact side demonstrated a mean of 26.2 ± 6.3 repetitions, whereas the injured side demonstrated a mean of 22.7 ± 5.8 repetitions. The median limb symmetry index was 87.5% (IQR, 81.4–93.3; range, 61.1–100.0%). The demographic, clinical, and functional characteristics of the study population are summarized in Table 1.

Based on the predefined 90% LSI threshold, 31 of 76 patients (40.8%) were classified as having sufficient functional recovery, whereas 45 patients (59.2%) had an LSI < 90%. The injured-side HRT performance was significantly higher in the LSI ≥ 90% group than in the LSI < 90% group (24.4 ± 5.4 vs. 21.6 ± 5.9 repetitions, *p* = 0.039). In contrast, ATRS scores were similar between the groups. Although the AOFAS score tended to be higher in the LSI ≥ 90% group, this difference did not reach statistical significance (100 [95–100] vs. 95 [88–100], *p* = 0.051). No significant differences were observed between the groups in demographic characteristics, follow-up duration, ankle range-of-motion deficits, calf atrophy, or postoperative complications. The comparison of patients by LSI group is summarized in Table 2.

**Figure 2:**
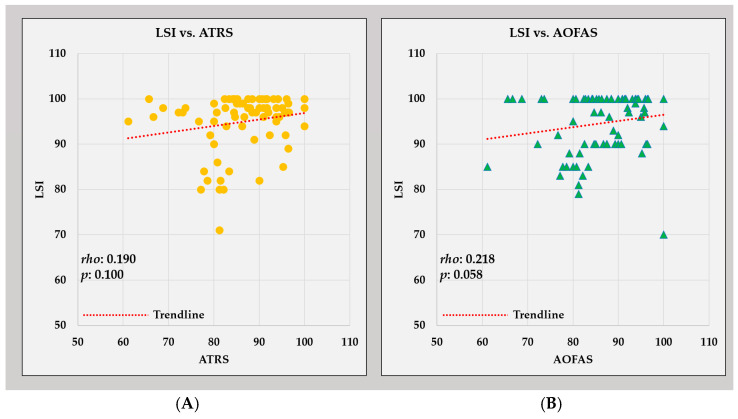
Scatterplots illustrating the relationship between limb symmetry index (LSI) and functional outcome scores. (**A**) LSI versus Achilles Tendon Total Rupture Score (ATRS). (**B**) LSI versus American Orthopaedic Foot and Ankle Society (AOFAS) Score. The dotted lines represent linear trend lines. No statistically significant correlation was observed between LSI and ATRS (Spearman’s rho = 0.190, *p* = 0.100). AOFAS showed a weak positive but statistically non-significant association with LSI (Spearman’s rho = 0.218, *p* = 0.058).

Furthermore, no significant correlation was found between LSI and either ATRS (Spearman’s rho = 0.190, *p* = 0.100) or AOFAS score (Spearman’s rho = 0.218, *p* = 0.058) (Figure 2).

A multivariable linear regression analysis was performed with heel-rise LSI as the dependent variable. After adjustment for age, BMI, and follow-up duration, neither ATRS nor AOFAS was independently associated with LSI. The regression coefficient was 0.201 for ATRS (95% CI, −0.242 to 0.643; *p* = 0.369) and 0.080 for AOFAS (95% CI, −0.325 to 0.486; *p* = 0.694). Age, BMI, and follow-up duration were also not significantly associated with LSI. The model’s overall explanatory power was low (R^2^ = 0.043), supporting the finding that patient-reported outcome scores did not adequately explain objective heel-rise limb symmetry (Table 3).

## 4. Discussion

In evaluating recovery after Achilles tendon repair, patient-reported outcome measures (PROMs) and objective clinical measurements represent complementary components of postoperative assessment [2]. PROMs, particularly scales such as the ATRS and AOFAS, are useful for reflecting patients’ perceived improvement in daily living activities and satisfaction with treatment [4,5]. However, these subjective scores may not fully reflect actual functional capacity and may overestimate recovery [2,8,9]. The literature indicates that patients may experience persistent deficits in strength and endurance despite high PROM scores, underscoring the importance of evaluating subjective satisfaction alongside objective performance [2,8,11].

Compared with similar studies in the literature, the ATRS and AOFAS scores in our study are consistent with the overall success rates reported after surgical repair [12,14,16]. For example, studies by Olsson et al. have shown that postoperative ATRS scores are generally high and that functional outcomes are favorable [12,13,14,16]. This suggests that the subjective satisfaction level in our cohort is consistent with the global literature. [17] However, the AOFAS score has been recognized to have methodological limitations and may exhibit a ceiling effect, potentially masking mild residual functional deficits [6,18].

Our findings also support this view; although our patients reported good-to-excellent PROM scores, these high scores did not necessarily indicate complete functional symmetry. Despite numerically lower ATRS and AOFAS scores in patients with LSI < 90%, no statistically significant differences were observed between groups. This finding suggests that PROMs may be insufficient to distinguish patients with residual objective functional deficits. However, the lack of statistical significance should be interpreted cautiously, as it may also reflect limited statistical power rather than true equivalence between the groups. Mild LSI deficits may not be perceived during routine daily activities and may therefore coexist with very high PROM scores. However, such deficits may become clinically relevant during higher-demand activities that require repetitive plantar-flexion strength, fatigue resistance, running, jumping, occupational loading, or a return to sport. Thus, the discordance observed in this study suggests that PROMs and heel-rise LSI reflect complementary yet distinct dimensions of recovery: patient-perceived daily function versus objective, performance-based capacity [2,8,9].

Objective measures have increasingly played a critical role in postoperative patient follow-up in recent years [2]. In addition to traditional assessments such as joint range of motion and calf circumference measurements, isokinetic strength testing and functional endurance analyses provide quantitative information on rehabilitation success and functional recovery [11]. The relatively wide range of calf circumference differences observed in our cohort may reflect inter-individual variability, including differences in baseline muscle mass, rehabilitation adherence, postoperative activity level, or measurement-related factors. The use of objective measures allows clinicians to move beyond simply relying on the patient’s subjective perception of “feeling better” and to quantify the extent to which functional recovery approaches the pre-injury biomechanical level [2,8]. The heel-rise test is one of the most practical and valid objective tests for evaluating calf muscle function after Achilles tendon injury [7,11]. The repetition-based protocol used in our study is consistent with real-world clinical practice and can be easily implemented during routine follow-up [19]. Olsson and colleagues demonstrated that patients who could perform a heel rise in the early postoperative phase reported higher PROM scores [20]. However, our long-term results suggest that this relationship may weaken over time. The finding that patients reported high satisfaction scores despite persistent deficits in heel-rise capacity suggests that such functional deficits may be compensated for during daily activities but may remain clinically relevant under conditions requiring endurance, fatigue resistance, or high-performance activity [2,8].

Recent studies have emphasized the importance of objective measures in evaluating Achilles tendon healing [21]. As demonstrated by Silbernagel and colleagues, combining the heel-rise test with parameters such as endurance and heel-rise height provides a more sensitive approach for identifying functional deficits in the muscle–tendon unit [7]. The objective functional deficits observed in our study are consistent with the calf muscle strength deficits reported by Brorsson and colleagues, which may persist even into the second postoperative year [22,23]. The literature indicates that postoperative tendon elongation and calf muscle atrophy are associated with impaired objective functional performance, even when subjective scores are high. In this context, the objective data from our study support the concept of persistent functional deficits following Achilles tendon repair, as described in the literature [24,25]. Therefore, clinical success should be evaluated not only on the basis of complication-free healing but also in conjunction with the restoration of limb symmetry [10,16].

The limb symmetry index (LSI) quantitatively assesses functional recovery by comparing the affected and unaffected limbs [10,15]. In the literature, an LSI value of 90% or higher is commonly used as a threshold for adequate functional recovery [15]. However, the 90% LSI threshold should be interpreted as a practical, literature-based clinical benchmark rather than an absolute cutoff for complete recovery. Although this value is useful for identifying residual side-to-side functional asymmetry, it does not directly indicate biological tendon healing, tendon morphology, or full restoration of pre-injury performance capacity. In the present study, the median LSI remained below this threshold, highlighting a discrepancy between patients’ subjective perceptions of recovery and objective functional performance, consistent with previous reports [9]. This discrepancy suggests that LSI may serve as a clinically meaningful indicator because it objectively reflects functional recovery and limb symmetry [10,15]. Although changes in tendon morphology have been shown to be associated with functional performance and may influence clinical outcomes [20], LSI should be interpreted primarily as a practical functional measure rather than a direct surrogate for biological tendon healing [10,24].

The primary strength of this study is that it evaluated postoperative recovery using a comprehensive approach that included not only patient-reported outcome measures (ATRS and AOFAS) but also objective functional assessments, particularly the heel-rise test-based limb symmetry index (LSI). The use of the widely adopted 90% LSI threshold enabled quantification of the discrepancy between subjective satisfaction and objective functional performance [15]. Additionally, the use of standardized surgical techniques and rehabilitation protocols across the entire cohort minimized treatment-related variability, thereby enhancing data reliability.

This study has several limitations. First, its retrospective design and the absence of preoperative baseline functional data limited the ability to compare postoperative recovery directly with the pre-injury condition. The retrospective design and single-center setting may also have introduced selection bias. Patients were identified from hospital records and were included only if they could be contacted, agreed to participate, and were able to complete the final clinical and functional assessment. Therefore, patients with poorer outcomes, lower motivation, limited access to follow-up, or inability to perform functional testing may have been underrepresented. In addition, because this study was conducted at a tertiary referral center, the cohort may not fully reflect the broader population of patients treated for acute Achilles tendon rupture in community-based or multicenter settings. Referral patterns, patient activity level, treatment preferences, rehabilitation access, and clinical complexity may differ in tertiary care settings, potentially affecting both PROMs and objective functional outcomes. Second, unmeasured confounding factors or explanatory variables not included in the present analysis may have influenced the findings. Although the multivariable analysis was adjusted for age, BMI, and follow-up duration, other potentially relevant factors, such as pre-injury activity level, rehabilitation adherence, tendon elongation, calf muscle strength, psychological factors, occupational demands, and return-to-sport status, were not available in this retrospective dataset. Therefore, the absence of an independent association between PROMs and heel-rise LSI should be interpreted with caution, as including these variables could lead to different interpretations of the relationship between subjective and objective recovery. Third, the study group consisted solely of male patients because the number of female patients in the eligible cohort was very small. Although this approach reduced sex-related heterogeneity in calf circumference, muscle strength, and heel-rise performance, it also limits the generalizability of the findings to female patients and may have introduced selection bias. Fourth, calf atrophy was assessed using a single-point calf circumference measurement. Although this measurement is simple, inexpensive, and clinically practical, it is only a crude proxy for muscle atrophy and does not capture muscle volume, muscle quality, fatty infiltration, contractile properties, or compartment-specific changes in the gastrocnemius–soleus complex. Therefore, calf circumference should be interpreted as an approximate indicator of side-to-side differences in calf size rather than a comprehensive assessment of muscle morphology or functional capacity. Finally, although the median follow-up duration of 41 months was sufficient to evaluate mid-term functional outcomes, longer-term prospective multicenter studies including broader patient populations, imaging-based muscle assessment, and objective strength testing are needed to more comprehensively assess persistent functional deficits, re-rupture risk, and the biological adaptation of the repaired tendon.

## 5. Conclusions

In conclusion, patient-reported outcome measures demonstrated high scores after surgical repair of acute Achilles tendon rupture; however, objective functional assessments revealed persistent deficits in limb symmetry. These findings suggest that PROMs may not fully capture objective functional recovery. Therefore, PROMs should be interpreted alongside performance-based measures to provide a more comprehensive evaluation of postoperative outcomes, particularly in patients with high functional demands.

## Figures and Tables

**Table 1:** Demographic, Clinical, and Functional Characteristics of the Study Population.

Variable	Unit	Value	Range
Age	years	42 (35–46)	27–74
Height	cm	176.7 ± 7.1	160.0–193.0
Weight	kg	85 (80–92)	59–125
BMI	kg/m^2^	27.3 (26.6–28.8)	21.4–38.6
Follow-up	months	41 (26–53)	12–80
Intact side HRT	repetitions	26.2 ± 6.3	8.0–40.0
Injured side HRT	repetitions	22.7 ± 5.8	7.0–38.0
LSI	%	87.5 (81.4–93.3)	61.1–100.0
ATRS	points	97 (94–99)	71–100
AOFAS	points	98 (90–100)	70–100
Dorsiflexion loss	degrees	0 (0–0)	0–10
Plantar flexion loss	degrees	0 (0–0)	0–20
Calf atrophy	cm	1.0 (1.0–2.0)	0.0–10.0

Abbreviations: BMI: Body Mass Index, HRT: Heel Rise Test, LSI: Limb Symmetry Index, ATRS: Achilles Tendon Rupture Score, AOFAS: American Orthopaedic Foot and Ankle Society. Values are presented as mean ± standard deviation for normally distributed variables and median (interquartile range) for non-normally distributed variables. Range is presented as minimum–maximum. Normality was interpreted using the Shapiro–Wilk test.

**Table 2:** Comparison of Patients According to Limb Symmetry Index Group.

Variable	Unit	LSI Low < 90% *n* = 45	LSI High ≥ 90% *n* = 31	*p* Value
Side, right/left	n	22/23	17/14	0.647 ^1^
Age	years	41 (35–46)	42 (36.5–46)	0.731 ^2^
Height	cm	176.3 ± 6.8	177.1 ± 7.7	0.636 ^3^
Weight	kg	85 (80–92)	86 (81–91.5)	0.571 ^2^
BMI	kg/m^2^	27.2 (26.8–28.7)	27.7 (25.9–28.9)	0.937 ^2^
Follow-up	months	39 (26–54)	42 (27.5–52)	0.601 ^2^
Intact side HRT	repetitions	26.5 ± 6.7	25.9 ± 5.9	0.706 ^3^
Injured side HRT	repetitions	21.6 ± 5.9	24.4 ± 5.4	0.039 ^3^
LSI	%	82.5 (79.2–85.7)	93.8 (91.7–96)	<0.001 ^2^
ATRS	points	97 (92–99)	97 (95.5–99.5)	0.318 ^2^
AOFAS	points	95 (88–100)	100 (95–100)	0.051 ^2^
Dorsiflexion loss	degrees	0 (0–0)	0 (0–0)	0.246 ^2^
Plantar flexion loss	degrees	0 (0–0)	0 (0–0)	0.482 ^2^
Calf atrophy	cm	1 (1–2)	1 (1–2)	0.834 ^2^
Any complication	*n* (%)	6 (13.3%)	2 (6.5%)	0.460 ^1^
Superficial wound infection	*n* (%)	5 (11.1%)	1 (3.2%)	0.391 ^1^
Sural nerve sensory disturbance	*n* (%)	1 (2.2%)	1 (3.2%)	1.000 ^1^

Abbreviations: BMI: Body Mass Index, HRT: Heel Rise Test, LSI: Limb Symmetry Index, ATRS: Achilles Tendon Rupture Score, AOFAS: American Orthopaedic Foot and Ankle Society. ^1^ Fisher’s exact test, ^2^ Mann–Whitney U test, ^3^ Independent samples *t*-test.

**Table 3:** Multivariable Linear Regression Analysis for Factors Associated with Heel-Rise Limb Symmetry Index.

Variable	β Coefficient	95% CI	*p* Value
ATRS	0.201	−0.242 to 0.643	0.369
AOFAS	0.080	−0.325 to 0.486	0.694
Age	−0.032	−0.245 to 0.180	0.764
BMI	0.172	−0.519 to 0.862	0.622
Follow-up duration	0.015	−0.098 to 0.129	0.788

Abbreviations: ATRS, Achilles Tendon Total Rupture Score; AOFAS, American Orthopaedic Foot and Ankle Society; BMI, Body Mass Index; LSI, Limb Symmetry Index; CI, confidence interval. LSI was entered as the dependent variable.

## Data Availability

The datasets are not publicly available. The de-identified data are available upon request from the corresponding author due to privacy, ethical, and legal restrictions that protect patient confidentiality.

## Abbreviations

The following abbreviations are used in this manuscript:

AOFAS	American Orthopaedic Foot and Ankle Society
ATR	Achilles tendon rupture
ATRS	Achilles Tendon Total Rupture Score
BMI	Body Mass Index
HRT	Heel-Rise Test
IQR	Interquartile Range
LSI	Limb Symmetry Index
PROMs	Patient-Reported Outcome Measures
SD	Standard Deviation
STROBE	Strengthening the Reporting of Observational Studies in Epidemiology
